# News in pharmacology for the main medical pathologies of gestation

**DOI:** 10.3389/fphar.2023.1240032

**Published:** 2024-01-04

**Authors:** María De Guadalupe Quintana-Coronado, Coral Bravo, Melchor Álvarez-Mon, Miguel A. Ortega, Juan A. De León-Luis

**Affiliations:** ^1^ Department of Public and Maternal and Child Health, School of Medicine, Complutense University of Madrid, Madrid, Spain; ^2^ Department of Obstetrics and Gynecology, University Hospital Gregorio Marañón, Madrid, Spain; ^3^ Health Research Institute Gregorio Marañón, Madrid, Spain; ^4^ Department of Medicine and Medical Specialities, Faculty of Medicine and Health Sciences, University of Alcalá, Alcalá de Henares, Spain; ^5^ Ramón y Cajal Institute of Sanitary Research (IRYCIS), Madrid, Spain; ^6^ Immune System Diseases-Rheumatology and Internal Medicine Service, University Hospital Príncipe de Asturias, Centro de Investigación Biomédica en Red en Enfermedades Hepáticas y Digestivas (CIBEREHD), Alcalá de Henares, Spain

**Keywords:** pharmacology, pregnancy complications, analgesia, antibiotic therapy, fetal maternal well-being

## Abstract

Obstetric diseases represent a highly complex medical challenge, especially regarding its clinical approach. The use of pharmacological agents during pregnancy is one of the main therapeutic alternatives in this group of patients; however, there is a general lack of knowledge about its use, efficacy, and possible adverse effects that may occur in routine clinical practice, even among medical professionals themselves. The high percentage of pregnant women who undergo drugs at some point during pregnancy, together with the developments that have occurred in recent years in the field of pharmacology, show the need for a detailed analysis that shows the existing current knowledge and helps in the clinical decision making. In this sense, the aim of this work is to conduct a review of the available scientific literature on the novelties in pharmacology for the main medical pathologies of pregnancy. Thus, the role of this field in analgesia, antibiotic therapy, digestive, respiratory, urological, psychiatric and neurological pathologies will be detailed, evaluating the indications, precautions and considerations that must be taken into account for its use.

## 1 Introduction

The objective of this work is to summarize the evidence in the literature on the novelties in pharmacology for the main medical pathologies of pregnancy. Approximately 80% of pregnant women are exposed to a drug during pregnancy (Lupattelli et al., 2014). The percentage of congenital malformations have risen to 1%–3%, which in part may be linked to maternal drug use. This range is even higher when some drugs, such as methotrexate, warfarin or retinoids, are used (Ito, 2016; de Waard et al., 2019). Additionally, there is a collective lack of knowledge about the consequences of drug use in pregnancy. In general, pregnant women are excluded from clinical trials, so the level of scientific rigor is low for this group of patients (Mitchell, 2003; Andrade et al., 2012). In addition, many of the studies carried out in these patients are inconclusive or are contradictory in terms of pharmacological risks and benefits (Colvin et al., 2010; Payne, 2019). That is why medical professionals in primary care and obstetrics & gynecology may lack concrete information on what to do when a subsidiary pathology requiring pharmacological treatment occurs during pregnancy.

We here aim to summarize current knowledge on the novelties in pharmacology in relation to the main medical pathologies encountered during pregnancy. In this work, the topics are presented under the following categories: analgesia, antibiotic therapy, digestive, respiratory, urological, psychiatric and neurological pathologies.

An electronic search was carried out through different databases, PubMed, UpToDate, Cochrane Library, Academic Google and Crossref, and relevant articles published in English and Spanish in the last 10 years were included. The keywords used in the search engine depended on the section in question but always included the phrase “during pregnancy”. Articles of potential interest were read and subsequently selected for inclusion in this review. Bibliographies of relevant articles were also checked for additional articles.

First, in 2015 the FDA (Foods and Drug Administration) replaced the former pregnancy risk letter categories on prescription and biological drug labeling with new information to make them more meaningful to both patients and healthcare providers. The A, B, C, D and X risk categories, in use since 1979, are now replaced with narrative sections and subsections. This recent labeling format known as The Pregnancy and Lactation Labeling Final Rule (PLLR) went into effect on June 30, 2015; however, the timelines for implementing this new information on drug labels is variable (Food and Drug Administration, 2014). The new sections include Pregnancy, Lactation and Females and Males of reproductive potential (Table 1). The pregnancy subsection contains a registry that collects data on pregnant women and notes any potential risks of medication use to the mother and the developing fetus. The lactation subsection contains information regarding the timing of breastfeeding, excretion of drugs in breastmilk, and risks to the infant. Finally, the females and males of reproductive potential subsection include relevant clinical information and recommendations about fertility, miscarriage, contraception, and pregnancy testing.

**TABLE 1:** Sections and subsections of the Pregnancy and Lactation Labeling Final Rule (PLLR) according to FDA.

The pregnancy and lactation labeling final rule (PLLR)
Pregnancy (includes Labor and Delivery)
Pregnancy Exposure Registry
Risk Summary
Clinical Considerations
Data
Lactation (includes Nursing Mothers)
Risk Summary
Clinical Considerations
Data
Females and Males of Reproductive Potential
Pregnancy Testing
Contraception
Infertility

## 2 Analgesia

Paracetamol or acetaminophen is generally considered the analgesic of choice for pregnant women with pain or fever (Bauer et al., 2021). However, recent studies have reported a possible risk associated with its continued use, related to neurodevelopmental (ADD, behavioral disorders), reproductive and urogenital tract problems (hypospadia, germ cell tumors) (Bauer et al., 2018; Konkel, 2018), and with greater risk in cases exceeding 28 consecutive days of treatment (Brandlistuen et al., 2013). That is why the current trend, based on a critical review of the literature that includes various cohort studies, is to recommend its prescription for the shortest possible time and the lowest effective dose while remaining the first-line analgesic treatment (Andrade, 2016) (Level of evidence I; 2++. Grade of recommendation B).

Regarding the use of NSAIDs, there is little agreement in the literature regarding their safety, and increased risks of abortion cardiac malformations or gastroschisis in newborns cannot be ruled out. However, the cohort studies supporting this idea have the limitation of not reporting the dose or exposure time (Nielsen et al., 2001; Nakhai -Pour et al., 2011), while those that describe a specific and controlled exposure deny such an association, except for with the use of indomethacin (16). However, all studies are in agreement regarding the increased risk associated with closure of the ductus arteriosus and fetal pulmonary hypertension if administered after week 28 (Van Marter et al., 1996; Van Marter et al., 2012) (Level of evidence II-2; 2+. Grade of recommendation C). In addition, in 2020, the FDA published an alert indicating that the use of NSAIDs from gestational week 20 was associated with kidney problems and oligohydramnios. The conclusion, therefore, is the possibility of prescribing NSAIDs in cases of uncontrolled acute pain at the beginning of pregnancy, always as the second choice and with ibuprofen, dexketoprofen and naproxen preferred over indomethacin and COX-2 inhibitors (Dathe et al., 2018) (Level of evidence II-2; 2+. Grade of recommendation C).

Metamizole can be used to control acute pain during the first trimester of pregnancy as a third-line treatment once use of the first and second drugs (paracetamol and ibuprofen) has been exhausted (Dathe et al., 2017) (Level of evidence II-2; 2+. Grade of recommendation C). However, its use is discouraged after gestational week 28 since it is related to alterations in fetal platelet aggregation and premature closure of the ductus arteriosus. A recent critical review describes the association with agranulocytosis of the newborn among breastfed infants whose mothers receive this treatment for a long time (Lampl and Likar, 2014) (Level of evidence I; 2++. Grade of recommendation B).

The risk of the use of aspirin or salicylate depends on the dose administered, with 150 mg being the recommended dose during pregnancy (Marhofer et al., 2021) (Level of evidence III; 4. Grade of recommendation D). Based on a randomized clinical trial, its use is recommended as a preventive measure for preeclampsia and its complications, as well as to prevent recurrent abortion (Rolnik et al., 2017) (Level of evidence I; 2++. Grade of recommendation B). Doses greater than 300 mg are associated with fetal heart disease and asthma, and doses greater than 650 mg are associated with maternal bleeding and intracranial hemorrhage in newborns (Black et al., 2019).

Exposure to opioids has generally been associated with the risk of withdrawal syndrome in newborns. However, its controlled use in acute pain is associated with a low risk of side effects that can be admitted based on the benefit provided to the pregnant woman (Patrick et al., 2015). Two systematic reviews of the literature report that short doses of pethidine, codeine, oxycodone, fentanyl or morphine are the best choice (Nagpal and Rathmell, 2013; Nunes et al., 2017) compared to the use of tramadol or trapentadol, since there are no studies that demonstrate their safety in relation to perinatal outcomes (Bloor et al., 2012) (Level of evidence I; 1+. Grade of recommendation B).

The use of antiepileptic drugs during pregnancy is usually reserved for patients with a diagnosis of epilepsy. However, according to the opinion of experts, there is the possibility of using antiepileptic drugs to control chronic pain in pregnant women with severe migraine or neuropathic pain (trigeminal neuralgia, diabetic neuropathy, fibromyalgia) that is difficult to control (Sardar et al., 2016) (Level of evidence III; 4. Grade of recommendation D). A systematic review recommends the use of gabapentin, followed by pregabalin, topiramate, levetiracetam or lamotrigine, instead of valproate or carbamazepine, since the latter are associated with neural tube defects and fetal neurodevelopment (Mølgaard -Nielsen and Hviid, 2011; Hernández -Díaz et al., 2012) (Level of evidence I; 2++. Grade of recommendation B). This review also recommends increasing the dose of folic acid to 5 mg during the first trimester and 1 month before conception (Ray-Griffith et al., 2018) (Level of evidence I; 2++. Grade of recommendation B).

Finally, the use of antidepressant treatment as a chronic analgesic in pregnant women has been sparsely studied (Payne, 2017). According to the opinion of experts, in pregnant women diagnosed with depression, treatment with SSRIs may be related to a minimal increase in fetal congenital malformations, but it is not clinically significant (Kennedy, 2013) (Level of evidence III; 4 Grade of recommendation D). The current recommendation, based on a critical review of the recent literature, is to limit its prescription to the lowest dose and time possible in poorly controlled patients with first-line analgesic drugs, as well as advising against abrupt suspension in patients with prescribed treatment prior to pregnancy. In the case of suspension, it is recommended to reduce the dose gradually and always under strict control of psychological symptoms (Payne, 2017) (Level of evidence I; 2++. Grade of recommendation B).

In conclusion, the current literature seems to advocate for a multidisciplinary approach to pain, with the first step being hygienic-dietary measures, such as weight gain control, healthy eating and lifestyle, as well as good postural hygiene (Wang et al., 2004). In the case of acute pain, the use of paracetamol is recommended, followed by traditional NSAIDs (ibuprofen) until week 20, and opioids (codeine) may be used if there is poor control of the symptoms. In pregnant women with disabling chronic pain (e.g., inflammatory, migraine, fibromyalgia), the use of paracetamol and NSAIDs is recommended, with the possibility of adding antiepileptic therapy (gabapentin) as an adjunct. The use of opioids or antidepressants could be evaluated as a third line, always with very strict control and using the minimum effective dose (Table 2).

**TABLE 2:** Analgesia conclusions during pregnancy.

Acute pain	Chronic pain
1. Hygienic-dietary measures	1. Hygienic-dietary measures
2. Postural measurements	2. Postural measurements
3. Paracetamol	3. Paracetamol
4. NSAID* (>metamizole) until week 20–28	4. NSAID* (>metamizole) until week 20–28
5. Opioids **	5. Antiepileptics ***
	6. Opioids/antidepressants ****

* Drug of choice: ibuprofen, naproxen, dexketoprofen

** Drug of choice: low doses of pethidine, codeine, oxycodone, fentanyl

*** Drug of choice: gabapentin, pregabalin, levetiracetam, topiramate, lamotrigine

**** Drug of choice: SSRI antidepressants

## 3 Antibiotherapy

In general, the use of antimicrobials is considered safe during pregnancy, and the benefit of treating an infection is greater than the risk posed by exposure to these drugs (Chow and Jewesson, 1985).

The critical review of observational studies carried out by Bookstaver et al. on antibiotic therapy during pregnancy indicates that the use of beta-lactams, metronidazole, clindamycin and fosfomycin is the choice over quinolones, macrolides or tetracyclines (Bookstaver et al., 2015) (Level of evidence I; 2 ++. Grade of recommendation B).

Various studies based on the opinion of experts describe some peculiarities of the antibiotics mentioned in the following lines:

- Among beta-lactams, there is talk of the essential role of penicillin as the first line of infection treatment in pregnant women, to the point of recommending desensitization therapy in patients with allergies and a diagnosis of syphilis (Lamont et al., 2014) (Level of evidence III; 4. Grade of recommendation D).
- Aminoglycosides (gentamicin, amikacin) have been associated with irreversible congenital bilateral deafness of the newborn, for which, in general terms, their use in pregnant women is discouraged (Briggs, 2014) (Level of evidence III; 4. Grade of recommendation D).
- Macrolides (erythromycin, better than clarithromycin or azithromycin) were previously associated with cardiovascular defects and pyloric stenosis, but a recent cohort study has disproved this association (Bahat Dinur et al., 2013) (Level of evidence I; 2+. Grade of recommendation B).
- The use of fluoroquinolones is related to cartilage damage, so it is recommended to avoid their use (Briggs, 2014) (Level of evidence III; 4. Grade of recommendation D).
- There is little information on glycopeptides (vancomycin), but the risk of nephrotoxicity or fetal deafness associated with their use cannot be ruled out, the risk being lower during the second and third trimesters (Medical Letter, 2021) (Level of evidence III; 4. Grade of recommendation D.
- Tetracyclines present a risk of teratogenicity, are contraindicated from week 5 of gestation and are associated with staining of teeth and defects in fetal bone growth (Smilack, 1999) (Level of evidence III; 4. Grade of recommendation D).
- Finally, clindamycin is generally considered safe until week 32, but its administration vaginally is discouraged due to the risk of premature delivery (Centers for Disease Control and Prevention, 2015) (Level of evidence III; 4. Grade of recommendation D).

Regarding antifungal therapy, we have focused on the most frequent pathology subsidiary to treatment in pregnant women: vulvovaginal candidiasis (Bender et al., 2021; Cooke et al., 2022). Asymptomatic *Candida* treatment decreases the risk of preterm delivery, low birth weight, and neonatal candidiasis (Mendling and Brasch, 2012; Ang et al., 2022b). Currently, several systematic reviews of the literature agree that the therapy of choice is with topical azoles such as clotrimazole (1 ovule for 6 days or 2 ovules for 3 days), reserving topical therapy with imidazole or nystatin for 2 weeks for recurrent cases (Young and Jewell, 2001; Marcelo Pradenas, 2014) (Level of evidence I; 1 ++. Grade of recommendation A).

Expert opinion (Bagga and Arora, 2020) advises against the use of oral fluconazole during pregnancy, since it is associated with a higher risk of miscarriage and tetralogy of Fallot (Level of evidence III; 4. Grade of recommendation D). A randomized clinical trial advises against repeated doses of oral fluconazole during breastfeeding, considering a single dose of 150 mg to be safe (Bauters et al., 2002) (Level of evidence I; 1+. Grade of recommendation B). Finally, another clinical trial from 2022 includes the use of *lactobacillus* probiotics as a method to reduce recurrent vulvovaginal candidiasis in pregnant women (Ang et al., 2022a) (Level of evidence I; 1+. Grade of recommendation B).

## 4 Digestive pathology

### 4.1 Nausea and vomiting

According to a randomized clinical trial, once the hygienic-dietary measures and postural recommendations have been exhausted, the pharmacological approach to nausea and vomiting in pregnant women starts with treatment with pyridoxine (vit B6), with or without doxylamine (CARIBAN 2 tablets per day), constituting a level A recommendation (Slaughter et al., 2014; Koren et al., 2015) (Level of evidence I; 1+. Grade of recommendation B). If symptoms persist, the use of metoclopramide 10 mg or ondansetron 4 mg every 8 h was previously recommended, but a current critical review based on observational studies recommends adding another antihistamine therapy (dimenhydrinate or prochlorperazine 25 mg) with CARIBAN before adding these treatments (Committee on Practice Bulletins-Obstetrics, 2018) (Level of evidence I; 1+. Grade of recommendation B). This is because ondansetron has recently been associated with neural tube closure defects, especially in the first trimester (AEMPS, 2023). Additionally, we highlight the importance of ensuring good hydration of the patient at all times, as well as reporting a meta-analysis that has shown that the use of ginger as a nonpharmacological therapy reduces nausea symptoms during pregnancy (Viljoen et al., 2014) (Level of evidence I; 1 ++. Grade of recommendation A).

### 4.2 Gastrointestinal reflux (dyspepsia and heartburn)

Gastrointestinal reflux is a frequent pathology during pregnancy, and its treatment has also evolved in recent years (Colvin et al., 2010; Malfertheiner et al., 2012). For its approach, various cohort studies and critical reviews of the literature recommend a well-defined diagnostic algorithm. It is advisable to take hygienic dietary measures followed by treatment with antacids, preferably containing aluminum or magnesium and not calcium, since this ion crosses the placenta and can affect the fetus, giving rise to calcium-alkaline syndrome (Malfertheiner et al., 2015). Alginates could also be considered first line since they act locally and are considered safe during pregnancy (Strugala et al., 2012). Once these resources have been exhausted, they recommend the use of sucralfate as a local mucosal protector at a dose of 1 g every 8 h (Thélin and Richter, 2020). As a third-line therapy, treatment with histamine H2 antagonists is proposed (Ali et al., 2022). The use of famotidine or cimetidine is recommended over ranitidine due to an FDA alert in 2020 that reports a high dose of N-nitrosodimethylamine, a carcinogenic element, in the product (US Food and Drug Administration, 2020). Finally, it is safe to use proton pump inhibitors (PPIs), which are considered the last choice in the GER. The use of lansoprazole, pantoprazole or esomeprazole is recommended over omeprazole (Ali et al., 2022) (Level of evidence I; 1 ++. Grade of recommendation A).

### 4.3 Constipation

There have been no developments in the treatment of constipation in recent years. A critical review of observational studies recommends increasing the intake of fluids and fiber-containing foods, followed by bolus-forming laxatives (methylcellulose) or osmotic laxatives (lactulose) (Christie and Rose, 2007; Zielinski et al., 2015) (Level of evidence I; 2+. Grade of recommendation B). Another review advises against the use of prokinetic agents and emollients such as castor oils, since the latter are associated with fat malabsorption and premature uterine contractions (Body and Christie, 2016b) (Level of evidence I; 2 ++. Grade of recommendation B).

### 4.4 Diarrhea

As with constipation, there has been little change in the treatment of diarrhea in pregnant women (Bonapace and Fisher, 1998). It is important to take into account that its approach will depend on the causative etiology and is always based on good hydration and electrolyte replacement in the patient. To highlight this, the results of a case‒control study showed that loperamide was associated with an increase in the number of cesarean sections, placenta previa and macrosomia and that diphenoxylate and bismuth were contraindicated due to the risk of teratogenesis (Kallen et al., 2008) (Level of evidence II-2; 2+. Grade of recommendation B). A 2006 systematic review considered the use only of spasmolytics, such as dicyclomine, in patients with very severe symptoms (Thukral and Wolf, 2006) (Level of evidence I; 1+. Grade of recommendation B).

### 4.5 Miscellaneous

Hemorrhoids: A randomized clinical trial on hemorrhoids reports a recommendation of specific dietary habits and hygiene care as a first measure (72) (Level of evidence I; 1+. Grade of recommendation B). A critical review has indicated that venotonics, such as diosmin and hesperidin, could be used with caution (73) (Level of evidence I; 2++. Grade of recommendation B).

Intestinal worms: Although the specific use of anthelmintic therapy (mebendazole, albendazole, praziquantel) seems safe during pregnancy, there is no agreement in the current literature. The most recent critical review that we have found (2020) advocates public interventions in maternal health to reduce poverty and improve the health conditions of homes (Mohan et al., 2020) (Level of evidence I; 2++. Grade of recommendation B).

## 5 Respiratory pathology

### 5.1 Asthma

With regard to asthma, the risks associated with poor control of the disease are greater than those derived from the use of anti-asthma medication. Asthma itself is related to a higher percentage of preeclampsia, intrauterine growth restriction, congenital malformations and perinatal death, which decreases when the disease is treated correctly (Wang et al., 2020). For strict symptom control, a recommendation to carry out monthly check-ups of pregnant women has been made (Global Initiative for Asthma, 2018). Based on various clinical trials, cohort studies and case‒control studies, the use of inhaled glucocorticoids (especially budenoside) is considered safe (Hodyl et al., 2011), as are short-acting beta-agonists (salbutamol), which are safer than long-acting ones (salmeterol) (Eltonsy et al., 2011). Leukotriene antagonists (e.g., montelukast), low-dose theophylline, and anticholinergic therapy (ipratropium bromide) have not been associated with major fetal malformations either but are second-line treatments if symptoms do not resolve with first-line drugs since it can be associated with preterm delivery (Cavero-Carbonell et al., 2017) (Level of evidence I; 1+. Grade of recommendation B). Systemic corticosteroid therapy has been associated with increases in preterm delivery and preeclampsia, among other complications (Zhang et al., 2016; Wang et al., 2020). However, according to the Global Initiative for Asthma (GINA) and National Asthma Education and Prevention Program (NAEPP) Asthma guidelines, its use is accepted, mainly when symptoms are exacerbated, since the risk is greater if asthmatic symptoms are not well controlled. Therapy with prednisone, prednisolone and methylprednisolone is the preferred therapy over the use of dexamethasone and betamethasone, which cross the placenta at higher concentrations (Zhang et al., 2016; Global Initiative for Asthma, 2018) (Level of evidence I; 1-. Grade of recommendation D).

Finally, regarding biological therapy, two recent reports have studied the use of omalizumab or anti-Ig-E without showing an increase in the risk of fetal malformations. While we have not found studies in the literature that approve the use of anti-IL5 drugs (Bonham et al., 2018; Chambers et al., 2021) (Level of evidence III; 4. Grade of recommendation D).

### 5.2 Respiratory infections

In respiratory infections, in general, good symptom control is advocated, recommending the use of paracetamol 1 g every 8 h when fever, accompanied by antibiotic treatment if necessary.

In upper respiratory tract infections, the use of antibiotics is not recommended except for symptoms of sinusitis, in which case several hospital guidelines based on systematic reviews of the literature consider the therapy with amoxicillin/clavulanate 875/125 mg orally every 12 h for 5–7 days, among others beta-lactams or macrolides (Chow et al., 2012; Rac et al., 2018) (Level of evidence I; 2++. Grade of recommendation B).

If we suspect infection by influenza virus in pregnant women, the current evidence, based on critical reviews, considers therapy with oral oseltamivir 75 mg every 12 h for 5 days to shorten the duration of illness and lessen the likelihood of complications among those infected (Beigi et al., 2014) (Level of evidence I; 2++. Grade of recommendation B).

For pneumonia, treatment with azithromycin 500 mg once a day is recommended, followed by 250 mg 4 days; over other beta-lactams with a broader spectrum of action. If pneumonia is complicated or requires admission, ceftriaxone 1–2 grams IV can be administered (Sheffield and Cunningham, 2009; Rac et al., 2018) (Level of evidence I; 2++. Grade of recommendation B).

## 6 Urological pathology

### 6.1 Urinary tract infection/pyelonephritis

Urinary tract infections, ranging from asymptomatic bacteriuria to pyelonephritis, have been associated with an increased risk of prematurity and low birth weight. For this reason, urine culture screening is recommended in each of the trimesters, as well as when the pregnant woman consults for voiding symptoms (Szweda and Jóźwik, 2016).

In patients with asymptomatic bacteriuria/cystitis, the SEGO (Sociedad Española de Obstetricia y Ginecología) recommends treatment with oral fosfomycin 3 g in single doses, amoxicillin 500 mg (associated or not with clavulanate 125 mg) every 8 h for 7 days, cefuroxime acetyl 250 every 12 h for 3–7 days or cefixime 400 mg every 24 h for 3–7 days as the first line. It also accepts the use of nitrofurantoin 50 mg every 6 h for 5 days (maximum limited treatment of 7 days according to the AEMPS- Agencia Española de medicamentos y productos sanitarios-) and trimethoprim-sulfamethoxazole 400 mg every 12 h for 3 days (except in the first trimester of pregnancy) (Sociedad Española de Ginecología Obstetricia, 2021). (Level of evidence I; 1+. Grade of recommendation A).

According to this same reference, in pregnant women with recurrent UTIs, prophylaxis could be performed with cephalexin 125–250 mg/day or cefaclor 250 mg/day (Sociedad Española de Ginecología Obstetricia, 2021) (Level of evidence I; 1+. Grade of recommendation A).

Based on the opinion of experts, in the case of patients with pyelonephritis, treatment will depend on whether they require admission (Generalitat de Catalunya, 2018; Platte, 2021) (Level of evidence III; 4. Grade of recommendation D).

- If admission is not needed, treatment with 1 g IV ceftriaxone single dose and home follow-up with 200 mg cefixime every 12 h orally for up to 7 days is appropriate.
- If admission is needed, 1 g ceftriaxone is preferred every 24 h IV for 7 days.

### 6.2 Renal colic

According to two retrospective observational studies and according to the opinion of experts published to date, for the treatment of renal colic, abundant hydration, placement in lateral decubitus on the unaffected side and rest are recommended, accompanied by the analgesic sequence listed in the first section. However, it is understood that, as its prevalence is greater in the second and third trimesters of pregnancy (from week 28), the use of NSAIDs is discouraged, favoring treatment with paracetamol 1 gr IV and short-term opioids (oxycodone, morphine, pethidine, and fentanyl) (Stothers and Lee, 1992; Parulkar et al., 1998; Dobiesz and Robinson, 2018; Bohorquez-Rivero et al., 2023) (Level of evidence II-3; 2-. Grade of recommendation D).

It is also of interest to avoid the use of buscopan, which are generally used in renal colic due to their analgesic and smooth muscle dilator roles. According to the AEMPS, its use in pregnant women is associated with CNS depression and neonatal bleeding due to Vit K coagulation deficits (Cima, 2021) (Level of evidence I; 2++. Grade of recommendation B).

## 7 Psychiatric pathology

### 7.1 Depression

When noticing symptoms of depression in a pregnant woman, referral to a specialist in psychiatry is recommended (Committee on Obstetric Practice, 2015). However, there are some premises associated with the general treatment of this entity. A meta-analysis carried out in 2013 associated the lack of treatment for depression with prematurity, low birth weight and postnatal complications itself (Grigoriadis et al., 2013) (Level of evidence I; 1++. Grade of recommendation A). In the review carried out by Byatt N et al. all a year earlier, the recommendation was to assess the benefit of providing a psychotropic medication, taking into account that monotherapy at the lowest possible dose will be the preferred choice, as opposed to polypharmacy. In addition, in general, it is advisable to delay the start of psychotherapy to the second trimester and gradually decrease the dose in the last weeks before delivery (Byatt et al., 2012) (Level of evidence I; 2++. Grade of recommendation B).

In relation to the most widely used drugs, the SSRIs, the conclusion of a cohort study published in 2014 was that, with the exception of paroxetine, which was related to cardiac malformations if administered in the first trimester, SSRIs were not considered teratogens (Huybrechts et al., 2014a) (Level of evidence II-2; 2 ++. Grade of recommendation B). The rest of the drugs in this group have been associated with an increase in the autism spectrum, persistent pulmonary hypertension and postnatal adaptation syndrome (irritability, insomnia and tremors), with a very low absolute risk (Huybrechts et al., 2014b) (Level of evidence III; 3. Grade of recommendation D).

SNRI, trazodone and mirtazapine, in the same way and according to the opinion of experts published in the current literature, are not associated with congenital malformations but are associated with respiratory distress at birth and postnatal adaptation sdr (Becker et al., 2016). However, a relationship has been found between the consumption of bupropion in the first trimester and some minor congenital heart malformations (Becker et al., 2016) (Level of evidence III; 3. Grade of recommendation D).

### 7.2 Anxiety and insomnia

According to a meta-analysis published in 2018, the approach to anxiety and insomnia is essential and should begin with hygienic-dietary measures, such as yoga, meditation, and continue with maternal education and psychological support (Grigoriadis et al., 2018) (Level of evidence I; 1++. Grade of recommendation A). A critical review of the literature concludes that SSRI antidepressants are preferred as preventive therapy (Becker et al., 2016) (Level of evidence II; 3. Grade of recommendation B). If proper anxiolytic therapy is used, the drugs used will be benzodiazepines (Becker et al., 2016).

The Organization of Teratology Information Specialists recommends, whenever possible, avoiding the use of benzodiazepines, especially between the third and eighth weeks of pregnancy, due to their association with cleft lip and palate defects. They also advise its suspension in the weeks before delivery to avoid withdrawal syndrome in the newborn (Mother To Baby, 1994) (Level of evidence III; 4. Grade of recommendation D).

A systematic review of the literature by Qilkinson D et al. concludes that the most widely used drug is diazepam. However, the American Academy of Pediatrics advises the use of lorazepam and alprazolam against this, since it seems that they cross the placenta to a lesser extent and have a shorter half-life. In no case should triazolam or flurazepam be indicated (Shyken et al., 2019) (Level of evidence I; 1+. Grade of recommendation B).

A case‒control study carried out by Wang LH et al. showed an association between Zolpidem (analog of BZD) use and an increase in preterm births, small-for-gestational-age newborns, cesarean sections and sdr abstinence, so the recommendation was to avoid it during the final stage of pregnancy (Wang et al., 2010) (Level of evidence II-2; 2+. Grade of recommendation B).

To date, two systematic reviews have reported no association between melatonin consumption during pregnancy and perinatal adverse effects (Wilkinson et al., 2016; Vine et al., 2022) (Level of evidence I; 1+. Grade of recommendation A). A randomized clinical trial is taking place that seems to find a relationship between the consumption of melatonin and the risk of brain damage in the fetus (especially in premature babies before week 28) (Valerie, 2023) (Level of evidence I; 1+. Grade of recommendation B).

## 8 Neurological pathology

### 8.1 Migraine

First, a headache due to preeclampsia, which is characteristically accompanied by hypertension, proteinuria, or organ failure, should be ruled out initially for patients with headache who are more than 20 weeks into gestation. A 2015 systematic review associated the presence of migraine with cardiovascular events during pregnancy (Wabnitz and Bushnell, 2015) (Level of evidence I; 1++. Grade of recommendation A).

In patients diagnosed with migraine, it is common to observe a decrease in symptoms during pregnancy, a consequence of the increased levels of endogenous estrogens and opioids during pregnancy (Allais et al., 2019). However, some patients suffer acute outbreaks and exacerbations, so it is important to know the most up-to-date treatment algorithm (Calhoun, 2017).

Regarding the use of paracetamol, NSAIDs and opioids, the recommendations are similar to the rest of the pathologies of pregnancy, as reflected in the previous sections. Two critical reviews of the literature published in 2017 and 2018 advise associating therapy with paracetamol, 10 mg of metoclopramide, 30 mg of codeine or 40 mg of caffeine if monotherapy is insufficient (Fox, 2004; Lee et al., 2018) (Level of evidence I; 1 ++. Grade of recommendation B). Diverse literature published to date concludes that the use of triptans has not shown a risk in terms of major fetal malformations (Fox, 2004; Cunnington et al., 2009). Likewise, sumatriptan is considered the preferred choice over other triptans due to its more hydrophilic pharmacodynamics. Its administration of 5–25 mg by the intranasal route is recommended since it reduces the dose that reaches the fetus and the symptoms of nausea and gastric stasis (frequent symptoms in the first trimester) (Amundsen et al., 2016) (Level of evidence II-2; 2+. Grade of recommendation B).

Finally, as a preventive treatment, the current literature recommends good sleep hygiene and lifestyle habits (Fox, 2004; Cunnington et al., 2009; Amundsen et al., 2016; Calhoun, 2017; Lee et al., 2018) (Level of evidence I; 1++. Grade of recommendation A).

### 8.2 Epilepsy

Epilepsy is the second most common neurological disorder after migraine, and it requires medical treatment in almost all cases. The most recent literature agrees that the first step is to recommend good preconception planning and comprehensive medication control during pregnancy (Harden and Lu, 2019) (Level of evidence III; 3. Grade of recommendation D).

The antiepileptic drugs used can be classified into classic and new-generation drugs (Voinescu and Pennell, 2015; Harden and Lu, 2019; Universitat de Barcelona, 2021; Li and Meador, 2022).

Various reports published to date relate the use of classic antiepileptic drugs with more teratogenesis and lower tolerability (Voinescu and Pennell, 2015) (Level of evidence III; 3. Grade of recommendation D).

According to an Expert review carried out by Voinescu PE et al., valproic acid, an antiepileptic drug for generalized genetic epilepsy, has been associated with major (12% Associated Risk (AR)) and minor congenital malformations (spina bifida, anencephaly, cleft palate, cardiovascular and genitourinary defects, and psychomotor retardation). Its use is recommended if there are no other alternatives and always at doses lower than 600 mg per day. Carbamazepine, useful in focal seiures, is associated with neural tube anomalies with a less aggressive profile than valproic acid (AR 2.6%–5%) (Universitat de Barcelona, 2021; Li and Meador, 2022) (Level of evidence III; 3. Grade of recommendation D).

In the same way, phenytoin, used for focal seizures, is related to adverse effects in the mother, such as drowsiness, ataxia, tremor and cognitive alterations, and in the fetus, such as ventricular septal defects, hypospadia and clubfoot (AR 2.9%–6.4%). It is characteristically associated with *hydantoin syndrome* (palate defects, cleft lip, saddle nose, hypertelorism, digital hypoplasia and mental retardation). Phenobarbital is associated with Fallot tetralogy and other congenital malformations with an AR of 5.5%–6.5% (Universitat de Barcelona, 2021; Li and Meador, 2022) (Level of evidence III; 3. Grade of recommendation D).

For their part, new-generation drugs are considered safer and are the preferred choice during pregnancy (Voinescu and Pennell, 2015). Lamotrigine, topiramate, levetiracetam and gabapentin can be used in both focal and generalized epilepsies. The use of lamotrigine presents the lowest percentage of major malformations, with an absolute risk that ranges from 2.3%–2.9%, without increasing the relative risk compared to women without epilepsy. Topiramate is associated with cleft lip and palate (3.9%–4.3%). Levetiracetam (with a 0.7%–2.8% risk) is also safe, although there is little evidence on its use. Finally, gabapentin has not been associated with a significant increase in major congenital malformations (2.2%–3.3%) or cognitive alterations in the offspring (Universitat de Barcelona, 2021) (Level of evidence III; 3. Grade of recommendation D).

In this group of patients, the use of folic acid supplements, which would be 5 mg per day, 3 months before conception and throughout pregnancy, is of special importance (Li and Meador, 2022) (Level of evidence III; 3. Grade of recommendation D).

## 9 Discussion

In this review, we summarize the current knowledge in pharmacology during gestation, grouping the evidence in disease categories. Other articles in the literature provide similar information on the risks and safety of the use of certain drugs during pregnancy. The safety information provided does not differ substantially from ours, but a description of the teratogen effects is also displayed for every medication with known deleterious effect.

For most indications in pregnant women, drugs are available with adequate clinical experience supporting drug safety. Still, there is insufficient information available on the risks and safety of many treatments (prescription or over the counter) known or available to patients and their healthcare professionals.

Unfortunately, pregnant women are often not included in clinical studies to determine the safety of medications before they are marketed. It has been reported that a more active and prolonged post marketing surveillance period is required to determine the true risk to the fetus. When patients and care providers search for information on a certain drug or medication, there are a number of organisations that spread information globally via their websites, such as the Organisation of Teratology Information Specialists, LactMed, EuroMediCat, Lareb, Cybele, Motherisk programme, Embryotox or Reprotox. Some regulatory bodies such as the FDA and Health Canada have recognised the lack of robust quality information for medical professionals and patients with regard to the use of medication during reproductive life. Also, the European Board and College of Obstetrics & Gynaecology in their call for action defends the necessity of improving the registration of data by the implementation of a general register of medication use in European pregnant women, insists on preconception counselling and interdiciplinary consultation, especially in women with chronic health issues.

Some initiatives in order to achieve a better access to realiable information is the metaPreg Project. This consists on a real-time full-scale living meta-analyses relying on an open online dissemination platform (www.metapreg.org). This website provides evidence syntheses dedicated to the risk of drug use during pregnancy and aims to cover all pharmacological treatments and to maintain up-to-date results in real-time.

## 10 Conclusion

In summary, the prescription of drugs during pregnancy should be a consensual, conscious and informed act, since in very few cases, their use is completely harmless for the maternal-fetal duality.

More established drugs that have lower risks associated with their use will be considered the preferred choice, with its application always tailored to the specific situation of the patient. In general, drugs are prescribed at the lowest effective dose and for the shortest time possible, and the risk-benefit of exposure is assessed individually.

In addition, correct pregnancy planning is important, especially in women of childbearing age with chronic medical conditions that require medication adjustment. Likewise, follow-up is recommended in specialized consultations for patients with high gynecological-obstetric risk to help to more exhaustively control the pregnancy.

Finally, in line with what was stated at the beginning of the work, certain contradictions have been found regarding the safety of the use of some drugs. That is why we consider it necessary to create reliable and conclusive action protocols that allow healthcare professionals to make a safe and documented decision.

## Abbreviations

FDA, Foods and Drug Administration; PLLR, The Pregnancy and Lactation Labeling Final Rule; ADD, attention deficit disorder; NSAIDs, non steroids anti-inflammatory drugs; COX-2 inhibitors, cyclooxygenase-2; SSRIs, Selective serotonin reuptake inhibitors; PPIs, proton pump inhibitors; GER, gastroesophageal reflux; GINA, Global Initiative for Asthma; NAEPP, National Asthma Education and Prevention Program; SEGO, Sociedad Española de Obstetricia y Ginecología; AEMPS, Agencia Española de medicamentos y productos sanitarios; UTIs, urinary tract infection; CNS, central nervous system; SNRI, serotonin-norepinephrine reuptake inhibitor; BZD, Benzodiazepine; AR, Associated Risk.
